# Gingival Blood Vessels in Smokers and Nonsmokers: A Scoping Review

**DOI:** 10.1055/s-0045-1809913

**Published:** 2025-08-01

**Authors:** Aura Hemalinda Adelia, Siti Sopiatin, Amaliya Amaliya

**Affiliations:** 1 Faculty of Dentistry, Universitas Padjadjaran, Bandung, Indonesia; 2 Department of Periodontology, Faculty of Dentistry, Universitas Padjadjaran, Bandung, Indonesia

**Keywords:** gingival blood vessels, smokers, nonsmokers

## Abstract

Smoking is an independent risk factor for periodontal disease. Despite the severity of periodontal disease, smokers demonstrated reduced clinical signs of inflammation in gingivitis and periodontitis. The present review analyzed the gingival blood vessels between smokers and nonsmokers to obtain a more comprehensive overview. A scoping review was conducted based on the Preferred Reporting Items for Systematic Reviews and Meta-Analyses for Scoping Review guidelines, according to the inclusion and exclusion criteria that have been set. Articles were retrieved from five databases: PubMed, Web of Science, EBSCOhost, Scopus, and ScienceDirect. The article searches were performed using keywords ((smoke OR smoking) AND (gingival vessel OR gingival vascular OR gingival microvasculature OR gingival vascularity OR gingival blood vessel OR gingival flow)). From an initial pool of 217 articles, 10 were ultimately chosen for detailed analysis. This selection encompassed seven cross-sectional studies, two case–control studies, and one experimental study. According to the articles that have been reviewed, there were differences in gingival blood vessel density between smokers and nonsmokers in different periodontal conditions, namely, healthy periodontal tissue, gingivitis, and periodontitis. The differences were more pronounced when smokers had gingivitis and periodontitis compared with nonsmokers with the same diseases. Assessment of blood vessel distribution showed that small and medium-sized vessels were more prevalent in smokers, while large vessels were more common in nonsmokers. Additionally, the lumen of blood vessels in smokers was narrower compared with nonsmokers.

## Introduction

According to the World Health Organization (WHO), periodontal disease ranks second highest among oral health problems. 1 It is prevalent both in developed and developing countries and affects approximately 20 to 50% of the global population. 2 Periodontal tissue is connective tissue surrounding the tooth, consisting of four components including the cementum, periodontal ligament, alveolar bone, and gingival tissue. 3 The gingiva is rich in blood vessels to deliver blood supply to maintain the health of periodontal tissue by providing nutrients and oxygen and transporting waste substances from the tissue. 4 5

The function of gingival blood vessels are affected by several factors, including lifestyle and diet, systemic diseases, hormonal changes such as pregnancy and puberty, and central nervous system mechanisms. 6 7 8 9 Central nervous system mechanisms, in the form of peripheral vasomotor control, can lead to changes in gingival blood vessels, affecting blood supply. This phenomenon is known to be associated with smoking. 10

Smoking is one of the main risk factors that can affect both systemic and oral health problems. 11 12 13 According to the WHO, there are an estimated 1.3 billion people who smoke worldwide. 14 Most of the tobacco-related deaths and years lost to disability are due to these noncommunicable diseases. 15 Tobacco smoking kills more than 8 million people each year, including approximately 1.3 million nonsmokers who are secondhand smokers. 16 Smoking has been shown to increase the risk of cardiovascular disease, lung cancer, and a variety of other serious health conditions. 17 18 19 In addition, tobacco smoking is associated with oral health problems such as oral cancer, oral mucosal lesions, implant failure, salivary gland hypofunction, dental caries, and periodontal disease. 20

Despite the deterioration of periodontal tissue, smokers showed reduced signs of inflammation in inflamed periodontal tissues, such as gingivitis and periodontitis, a condition associated with vasoconstriction on the gingival blood caused by cigarette smoking. 21 22 Moreover, histological assessment revealed that smokers' gingiva experienced changes in capillary density, fibrosis, and gingiva epithelial thickness when compared with normal gingiva. 23 24 These changes cause smokers' gingiva more susceptible to infection and ongoing tissue damage and have a slower and less optimal healing response. 25 26 Given the significant impact on gingival health, it is important to perform a comprehensive review on gingival vasculature in smokers and nonsmokers. The present scoping review aims to examine the conditions of gingival blood vessels between smokers and nonsmokers.

## Methods

A scoping review was performed according to the protocol by the Joanna Briggs Institute 27 and the Preferred Reporting Items for Systematic Reviews and Meta-Analyses for Scoping Review. Article searches and analyses were conducted from October to December 2024. Five databases, that is, PubMed, Web of Science, EBSCOhost, Scopus, and ScienceDirect, were searched for keywords and Boolean operators as follows: ((smoke OR smoking) AND (gingival vessel OR gingival vascular OR gingival microvasculature OR gingival vascularity OR gingival blood vessel OR gingival flow)).

PCC (population, concept, context) guidelines were used to determine eligible articles. Population (P): smokers and nonsmokers; concept (C): gingival blood vessels in smokers and nonsmokers assessed by clinical and laboratory evaluation; and context (C): smokers and nonsmokers both female and male aged more than 18 years and above in all studies conducted in various countries contained in the publication. The inclusion criteria were articles with a study population of smokers and nonsmokers, articles that discussed the morphology of gingival blood vessels, articles with observational (cross-sectional and case–control study) and experimental research designs, full-text articles, articles in English, and articles with year published since the first publication of literature discussing the relationship between smoking and its effect on gingival blood vessels. Exclusion criteria included articles with animal subjects, literature reviews, and systematic review articles.

All articles were transferred and screened, and duplicates were eliminated using the Mendeley software. Articles were screened by title and abstract, and potential articles were selected. Further selection was made after reading the full text of potential articles according to the inclusion and exclusion criteria. Articles that met the inclusion criteria were included in this study and then data extraction was performed. The data information extracted is the author, year, title, research design, sample size, sample age, sample criteria, parameters, and study results.

## Results

**Fig. 1:**
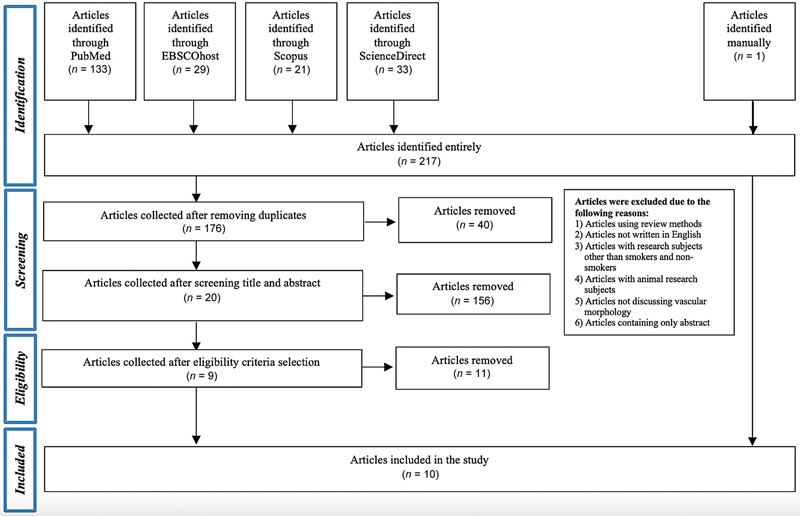
The Preferred Reporting Items for Systematic Reviews and Meta-Analyses for Scoping Review (PRISMA-ScR) flowchart of the search and selection process.

A total of 217 articles were identified from the databases, 40 were duplicates and removed. The title and abstract of 176 articles were assessed, and 156 were excluded. The remaining 20 underwent full-text examination, and then 10 studies were finally included in the review ( Fig. 1 ). 22 28 29 30 31 32 33 34 35 36

### Study Characteristics

The present review included articles with years of publication from the first publication of articles discussing the relationship between smoking and gingival blood vessels, with the first article published in 1987 and the last one published in 2020. The research designs used in the articles reviewed were cross-sectional, case–control, and experimental. Among the 10 studies related to this topic; 7, 2, and 1 were cross-sectional, case–control, and experimental studies, respectively.

A review of the research articles yielded data on the characteristics of the participants involved in the studies. A total of 530 individuals were involved in the studies in the selected articles consisting of smokers and nonsmokers. The smallest sample size in the selected articles was 14 and the highest subject number was 200. Participants involved were those with healthy gingiva, gingivitis, and periodontitis. Smoker subjects had a smoking experience of 5 to 25 cigarettes per day with an average duration of more than 5 years.

In the present review, parameters used in the articles studied were evaluated to investigate any differences in gingival blood vessels in smokers and nonsmokers, that is, vascular density (VD), distribution of small (SV), medium (MV), and large (LV) blood vessels, and lumen area or diameter of blood vessels. Out of 10 articles reviewed, 7 articles showed differences in gingival blood vessel density in smokers and nonsmokers. There were 4, 2, 1, 1, 1, and 1 articles that evaluated vascular density; VD, SV, MV, LV, and lumen area; VD and MV; SV and LV; the vascular distribution, such as SV, MV, and LV; and SV, respectively ( Table 1 ).

**Table 1:** Data presentation of gingival blood vessels between smokers and nonsmokers ( *n* = 10)

**No**	**Author (Years)**	**Title**	**Study design**	**Sample**					**Research time**	**Blood vessels measurement indicators**	**Methods and tools**	**Results**	**Conclusions**
				***n***	**Age** **(y)**	**Periodontal tissue conditions**	**Criteria**						
							**Smokers**	**Nonsmokers**					
1	Persson and Bergstro ¨m 28 (1998)	Smoking and vascular density of healthy marginal gingiva	Cross- sectional	14 (7 smokers and 7 nonsmokers)	25–38 (average 30.4)	Healthy	10 to 25 (mean 16.1) cigarettes per day and smoking duration ranged from 8 to 18 (mean 13.1) years.	No previous history of smoking	N/A	Vascular density (VD)	Photostereography	Smokers: Mean (SD) VD scores ranged from 7.57 (3.78) to 12.21 (8.62) Nonsmokers: VD scores of 10.71 (8.54) 11.71 (8.12) In nonsmokers over the 11-week study period	There is a difference in vascular density
2	Lindeboom et al 29 (2005)	Effect of smoking on the gingival capillary density: assessment of gingival capillary density with orthogonal polarization spectral imaging	Cross- sectional	20 (10 smokers and 10 nonsmokers)	Smokers: 25 ± 1.2 Nonsmokers: 25 ± 1.4	Healthy	10 cigarettes per day for at least 5 years.	People who have never smoked	N/A	Vascular density (VD)	Image capture with K7K software technology	Smokers: VD = 69.3 ± 8.9 vessels per visual field Non-Smokers: VD = 60.6 ± 5.4 vessels per visual field	There is a difference in vascular density
3	Bergström et al 30 (1988)	Influence of cigarette smoking on vascular reaction during experimental gingivitis	Experimental	16 (8 smokers and 8 nonsmokers)	19–42	Gingivitis	10 to 20 (average 13.4) cigarettes per day for at least 4 years	Never smoked	28 days	Vascular density (VD)	Stereoscopic monitoring	Day 14: The number of vessels in smokers was 26.2 ± 5.06, while in nonsmokers it was 50.1 ± 8.55 ( *p* < 0.05). Day 28: The number of vessels in smokers was 45.5 ± 7.50, while in nonsmokers it was 98.4 ± 18.2 ( *p* < 0.05).	There are differences in vascular density
4	Sreedevi et al 22 (2012)	Periodontal status in smokers and nonsmokers: a clinical, microbiological, and histopathological study	Case–control	200 (100 smokers and 100 nonsmokers)	25–50	N/A	12 subjects: < 1 pack year50 subjects: 1–5 *pack years* , 24 subjects: 5–15 *pack years* 14 subjects: had a history of smoking > 15 years.	Never smoked	N/A	1. Vascular density (VD) 2. Medium vessel distribution (MV)	Biopsy and staining with H&E	VD smokers: 8.84 ± 0.96 Nonsmokers: 11.12 ± 1.23 MV nonsmokers: 46 vessels Smokers: 39 vessels. Blood vessel diameters of 4–8 mm were significantly smaller in smokers ( *p* = 0.073), explaining the vasoconstrictive effect of smoking on few blood vessels	Differences in blood vessel density and distribution of medium blood vessels
**No**	**Author (years)**	**Title**	**Study design**	**Sample**					**Research time**	**Blood vessels measurement indicators**	**Methods and tools**	**Results**	**Conclusions**
				***n***	**Age** **(y)**	**Periodontal tissue conditions**	**Criteria**						
							**Smokers**	**Nonsmokers**					
5	Jalayer Naderi et al 31 (2015)	The impact of smoking on gingiva: a histopathological study	Case–control	60 (28 smokers and 32 nonsmokers)	20–60	Periodontitis	Duration of smoking > 3 years	Never smoked	N/A	Distribution of small blood vessel (SV)	Flap surgery and staining with H&E	Average number of blood vessels with diameter ≤ 0.5 μm: Smokers: 18.78 ± 10.06 Non-smokers: 5.90 ± 2.93	There are differences in the distribution of small vessels
6	Rifai ET AL 32 (2020)	Evaluation of the papillary gingival vasculature in smokers and nonsmokers with chronic periodontitis: a clinical in vivo study	Cross- sectional	20 (10 smokers and 10 nonsmokers)	30–60	Periodontitis	> 10 cigarettes/day for the past 10 years	Never smoked	N/A	Vascular distribution (SV, MV, LV)	Immunohistochemical staining with CD34 *mouse monoclonal body*	Biopsy results under the sulcular epithelium Smokers: SV: 6.80 (± 1.89) MV: 2.80 (± 1.48) LV: 0.50 (± 0.50) Nonsmokers: SV: 8.50 (± 2.29) MV: 2.30 (± 1.79) LV: 0.60 (± 1.34)	There are differences in the distribution of small, medium, and large vessels
7	Dayakar et al 33 (2015)	Histomorphometric analysis of gingival tissue in smokers and nonsmokers	Cross- sectional	30 (15 smokers and 15 nonsmokers)	N/A	Periodontitis	Average of 5–10 cigarettes per day for more than 2 years	Never smoked	N/A	1. Vascular density (VD) 2. Vascular distribution (SV, MV, LV) 3. Lumen area	*Biopsy and interpretation using ImageJ analyzer system*	Smokers VD = 7.27 ± 3.283; Average SV and MV = 4.0 ± 1.512 and 3.0 ± 1.927 ; LV = 1.47 ± 1.552 Nonsmokers VD = 8.6 ± 2.354; Average SV and MV = 2.4 ± 1.844 and 2.4 ± 1.298 ; LV = 4.0 ± 2.035 Mean lumen area and vascular density in smokers were smaller than in nonsmokers	There are differences in blood vessel density, distribution of small, medium, and large vessels, and lumen area
8	Kumar and Faizuddin 34 (2011)	effect of smoking on gingival microvasculature: a histological study	Cross- sectional	33 (18 smokers and 15 nonsmokers)	N/A	Periodontitis	> 10 cigarettes per day for more than 3 years	Never smoked	N/A	1. Vascular density (VD) 2. Vascular distribution (SV, MV, LV) 3. Lumen area	*Biopsy and interpretation using ImageJ analyzer system*	Smokers: VD = 12.388 ± 6.472 %SV = 83.9% %MV = 14.8% % LV = 1.3% Lumen area: 19.290 ± 8.775 μm ^2^ Nonsmokers VD = 14.800 ± 4.901 %SV = 82.9% %MV = 15.3% %LV = 1.8% Lumen area: 20.044 ± 7.896 μm ^2^	There are differences in blood vessel density, distribution of small, medium, and large vessels, and lumen area
**No**	**Author (years)**	**Title**	**Study design**	**Sample**					**Research time**	**Blood vessels measurement indicators**	**Methods and tools**	**Results**	**Conclusions**
				***n***	**Age** **(y)**	**Periodontal tissue conditions**	**Criteria**						
							**Smokers**	**Nonsmokers**					
9	Mirbod et al 35 (2001)	Immunohistochemical study of vestibular gingival blood vessel density and internal circumference in smokers and nonsmokers	*Cross-sectional*	17 (5 smokers and 12 nonsmokers)	28–69 (average 48.4 ± 12.4)	Periodontitis	20–30 cigarettes per day for at least 5 years	Never smoked	N/A	Vascular distribution (SV and LV)	CD34 *Immunohistochemical staining*	Smokers showed a higher proportion of small vessels (IC < 50 μm) and a lower proportion of large vessels (IC > 100 μm) which was statistically significant ( *p* = 0.0403 and *p* < 0.001, respectively) Insignificant difference in vascular density between smokers and nonsmokers	There are differences in distribution of small and large vessels
10	Prakash et al 21 (2014)	Comparative evaluation of the marginal gingival epithelium in smokers and nonsmokers: a histomorphometric and immunohistochemical study	*Cross-sectional*	120 (60 healthy smokers and periodontitis and 60 healthy nonsmokers and periodontitis)	< 55	Healthy and periodontitis	Smoking ≥ 10 cigarettes for at least 10 years	Never smoked	N/A	Vascular density (VD)	Biopsy, immunohistochemical staining, and histomorphometric analysis	VD: Healthy smokers: 310.76 (4.13) Periodontitis smokers: 412.13 (30.19) Healthy nonsmokers 340.07 (32.93) Periodontitis nonsmokers: 325.47 (29.45) Participants who smoked had fewer signs of inflammation, fewer vascular elements in the connective tissue layer of the subepithelial and significantly reduced blood vessel caliber compared with nonsmokers	There are differences in vascular density

Abbreviations: H&E, hematoxylin and eosin; N/A, not available; SD, standard deviation.

### Differences in Vascular Density of Smokers and Nonsmokers with Healthy Periodontal Tissues, Gingivitis, and Periodontitis

Numerous studies evaluated differences in vascular density between smokers and nonsmokers with healthy periodontal tissue, gingivitis, and periodontitis. 22 28 29 30 33 34 36 Based on the evaluation of periodontally healthy subjects, Persson and Bergström 28 showed that vascular density in nonsmokers was higher compared with smokers. However, there was no clear difference between the two groups. Meanwhile, in gingivitis state, Bergström et al 30 revealed that vascular density in smokers and nonsmokers increased as the study progressed, with vascular density in smokers relatively less than in nonsmokers. 30 The results of this study suggested that smoking led to vasoconstriction of blood vessels in the gingiva, thus inhibiting the increase in the number and size of blood vessels that occur during inflammation. Studies conducted by Sreedevi et al 22 and Jalayer Naderi et al 31 also support this theory, it was found that inflammation in smokers' gingiva was lower than in nonsmokers. 22 31 They suggested the narrowing of peripheral blood vessels was due to substances contained in cigarettes. This narrowing of blood vessels may reduce inflammation, such as bleeding, redness, and exudation. 22 Differences in gingival vascular density between smokers and nonsmokers were also found in populations with periodontitis. 33 34 36 Based on the results of a study by Dayakar et al, 33 it was found that the average gingival blood vessel density was higher in nonsmokers compared with smokers. The findings were in agreement with the results of a study by Kumar and Faizuddin, 34 which showed similar results.

### Differences in the Distribution of Small-Sized Vessel, Medium-Sized Vessel, and Large-Sized Vessel between Smokers and Nonsmokers

The distribution of small (SV), medium (MV), and large (LV) blood vessels in the gingiva also showed differences between smokers and nonsmokers. 22 31 32 33 34 35 Kumar and Faizuddin 34 and Dayakar et al 33 concluded that SV and MV were found to be more prevalent in smokers, while LV was higher in nonsmokers. These findings confirmed the study by Lindeboom et al 29 who reported that small blood vessel density is higher in smokers than nonsmokers. These results were obtained due to the different methods of the study, which observed microblood vessels or capillaries. In addition, it is also supported by Mirbod et al, 35 which stated that the proportion of SV is higher and LV is lower in smokers.

### Lumen Area Differences between Smokers and Nonsmokers

The lumen area measured by the Image-J analyzer was found to be narrower in smokers compared with nonsmokers. 33 34 It was due to vasoconstriction associated with nicotine in cigarettes that stimulates the production of adrenaline and noradrenaline. 31 As a result, smokers tended to have mild signs of inflammation, which in turn caused a reduction of redness and bleeding. 22 31

## Discussion

The present review comprehends vascular density, distribution of vessels, and gingiva vascular lumen area in smokers and nonsmokers with healthy periodontal tissue, gingivitis, and periodontitis. 21 22 28 29 30 31 32 33 34 35 Based on studies in periodontally healthy subjects, Persson and Bergström 28 showed that vascular density in nonsmokers was higher compared with smokers. However, there was no clear difference between the two study populations. The insignificant difference in vascular density between smokers and nonsmokers with healthy gingiva may be caused by several factors. It is likely that in healthy gingiva many neutrophils may help the healing process of tissues, including blood vessels. This causes blood flow and blood vessels to remain protected from exposure to harmful cigarette content. 37 However, it should be noted that smoking has an impact on the whole oral cavity. 20 38 Therefore, although in healthy gingiva the difference in vascular density is not significant, smoking still poses a high risk for long-term oral health. 20 38

On the other hand, in inflamed periodontal tissues, differences in vascular density of smokers versus nonsmokers tended to be more noticeable. 21 30 39 Bergström et al demonstrated that vascular density in nonsmokers with gingivitis increased as the disease progressed, nevertheless, smokers showed relatively less dense vasculatures than nonsmokers. 30 Not only in the gingivitis state but the difference in vascular density also appears in subjects with periodontitis. 21 33 34 Periodontal disease occurs due to interactions between infectious agents (bacterial plaque) and host factors. The first event in the vascular phase of inflammation is the increase of small blood vessels adjacent to the injury dilate (vasodilatation) and blood flow to the area increases. In smokers, it seems the vascular system cannot compensate and fails to respond properly to the irritation or stimulation. This is particularly due to changes in blood vessels in the gingiva that disguise signs of inflammation. 39 In periodontitis, periodontal tissue is undergoing chronic inflammation and escalating the body's need to increase local vascularization. 40 41 Unfortunately, in smokers who experience periodontitis, smoking inhibits the formation of new blood vessels and may lead to the narrowing of blood vessels, despite the body's efforts to increase blood flow to the infected area. 42 43

The distribution of small-sized vessels (SV) and medium-sized vessels (MV) appears to be more prevalent in smokers compared with nonsmokers. 31 33 34 35 This result is due to the body's response to chronic hypoxia caused by exposure to cigarette smoke. 44 This hypoxia is known to occur since carbon monoxide in cigarette smoke competes with oxygen to bind to hemoglobin, thereby reducing the amount of oxygen available to tissues. 45 As a compensatory response, the body increases angiogenesis or the formation of new blood vessels, stimulated by the release of growth factors such as vascular endothelial growth factor. 46 47 48 Angiogenesis is influenced by histological, biochemical, and physiopathological factors acting on embryonic structures. Endothelial progenitor cells (EPCs) play an important role in the process of angiogenesis, especially in supporting most of the healing and repair processes in damaged vascular systems. However, changes in the number, characteristics, or function of EPCs can negatively impact the angiogenesis process. Therefore, factors that may interfere with the optimal function of EPCs, such as smoking, have the potential to impair the body's angiogenic response. 49 In other words, although the distribution of small- and medium-sized blood vessels in smokers with periodontitis is greater than that of nonsmokers, these blood vessels may be suboptimal in their function.

Besides causing hypoxia, cigarette smoke can also impair nitric oxide (NO) production in the body, resulting in decreased NO levels and increased oxidative stress. 50 51 Conversely, exposure to cigarette smoke can increase endothelin-1 (ET-1) levels. 52 53 NO functions as a vasodilator, which dilates blood vessels, while ET-1 acts as a vasoconstrictor, which causes constriction of blood vessels. 54 55 ET is a much stronger substance than norepinephrine and angiotensin Il in constricting blood vessels. 55 The release of ET-1 can be triggered by various things, such as lack of oxygen (hypoxia), shear stress in blood vessels, harmful substances in the blood (endotoxins), or other factors such as epinephrine, angiotensin Il, and smoking. 56 In chronic smokers, ET-1 levels in the blood are upregulated. 55 Thus, if vasoconstriction occurs continuously, the proportion of large-sized blood vessels will also decrease.

Lumen area was also found to be narrower in smokers than in nonsmokers. 33 34 This is also caused by vasoconstriction that not only occurs due to the increase in ET-1 by cigarette smoke but also the nicotine content in cigarettes. Nicotine will trigger the release of adrenaline and noradrenaline. 57 These catecholamines will bind to α-1 receptors on vascular smooth muscle, which causes the muscle to contract and blood vessels to narrow (vasoconstriction) so that the diameter of the lumen of the blood vessels in smokers is narrower than in nonsmokers. 57 58 Other studies have reported acute vascular reactions to smoking, although the vasoconstrictive response will still occur in small intensity, the hyperemic response due to high blood pressure can overcome the vasoconstriction that occurs. Therefore, blood can still flow to the tissues around the gingiva in acute smokers. 56 59 Nicotine is often considered the main cause of the adverse health effects of smoking. Nicotine is not considered to be carcinogenic. 60 Smokers inhale nicotine, but are killed by tar. 60 61 In other words, the most harmful substance in cigarettes is tar. Tar is the carcinogenic and mutagenic residue of cigarette combustion, which is the main cause of the most harmful effects associated with smoking. 62 As a total result, cigarette smoking leads to structural alterations of different vascular beds, causes blood vessels to increase stiffness, tone, resistance, intima-media thickness, wall thickness, aneurysmal dilation, endothelial cell injury, and decrease compliance and elasticity of blood vessels, and it may end up in smaller size of lumen.

The amount and duration of smoking greatly influence the impact of smoking on periodontal tissues. A light smoker is defined as someone who smokes less than 1 pack per day, less than 15 cigarettes per day, less than 10 cigarettes per day, or between 1 and 39 cigarettes per week. 63 A moderate smoker is someone who smokes 11 to 20 cigarettes per day. 64 Meanwhile, a heavy smoker is defined as a smoker who smokes more than 25 cigarettes per day. 65 In this study, the average individual smoked 5 to 25 cigarettes per day with an average duration of more than 5 years. Heavy smokers were found to have vascular changes in their gingiva. Chronic narrowing of blood vessels due to smoking will lead to a low density of distributed blood vessels, resulting in reduced blood supply to surrounding tissues and decreased gingival crevicular fluid flow. 22 33 34 36 As a result, the inflammatory response will be masked (masking effect). 66 Constriction in the gingival blood vessels will suppress inflammatory clinical signs such as bleeding, redness, and exudation. 22 Despite the differences found in gingival vasculature between smokers and nonsmokers, it is important to recognize that smoking can also have negative effects, such as resulting in decreased local immunity, worsening plaque buildup, increasing the risk of infection, slowing wound healing, and worsening periodontal disease symptoms. 66 67 68 69

Numerous clinical investigations on how smokers and nonsmokers react to different forms of periodontal therapy, including both nonsurgical and surgical techniques, have been performed by researchers. 1 20 Since up to 90% of patients with refractory periodontitis have been found to smoke, it has been proven that smoking adversely affects all forms of periodontal therapy. 21 Following nonsurgical and surgical therapies, researchers have observed that nonsmokers have much lower probing depths, less blood, and improved clinical attachment. 22 23 When treating furcation lesions and following regeneration techniques, a comparable result has been noted. 24 25

Research indicates that compared with nonsmokers or former smokers, current smokers exhibit less notable changes in clinical responses following scaling and rootplaning (SRP), such as a decrease in probing pocket depth (PPD) and an increase in clinical attachment loss (CAL). 16 However, at 1 and 3 months after nonsurgical therapy, Jin et al 70 discovered that nonsmokers had much larger decreases in the order of 1.0 mm than smokers. 54 Additionally, based on a decrease in probing depth, an increase in clinical attachment levels, and fewer bleeding episodes following periodontal probing, a study by Poucher et al 71 found that after 9 months, nonsmokers and smokers experienced the same degree of relief from nonsurgical therapy. In addition, clinical research has also demonstrated that smokers have worse healing response outcomes after periodontal surgery and endodontic treatment than nonsmokers. 55 68 69 72

Pathogenesis of smoking-related differences in the diameter of blood vessels is a result of interaction between reactive oxygen species (ROS)-generating enzymes, such as mitochondrial oxidases, and NOXs as important sources of vascular ROS production and contributes to the initiation and maintenance of endothelial dysfunction, vascular inflammation, and vascular remodeling. 72 From a morphological aspect, initially, morphological alterations from smoking are caused by a mechanism due to hypoxia as a result of carboxyhemoglobin produced by carbon monoxide. On the microcirculation, tobacco smoking leads to compromised endothelial-dependent vasorelaxation, platelet aggregation, endothelial cell dysfunction, and the activation of circulating leukocytes. Through these mechanisms, cigarette smoking elicits the aggregation and adhesion of leukocytes and/or platelets to the microvascular endothelium in the venules and arterioles by the participation of ROS, inflammatory mediators, and adhesion molecules in the orchestration of microcirculatory dysfunction after cigarette smoking. 73

The findings of this scoping review show differences in the gingival vasculature of smokers and nonsmokers with healthy periodontal tissue, gingivitis, and periodontitis. This study has limitations as the majority of articles focused on cross-sectional research designs that conclude at a single point in time, and experimental studies were scarce. Some of the articles reviewed also had varying measurement methods, so the results cannot be considered uniform. In addition, the majority of the population in the articles reviewed had periodontal tissues that experienced periodontitis, while studies with populations with healthy periodontal tissues and gingivitis were still limited. Therefore, further research involving more diverse populations, more specific study designs and measurement methods, as well as more in-depth exploration of other parts of the gingiva, is needed to strengthen the existing evidence.

## Conclusion

Based on the present review, there were differences in gingival vascular density between smokers and nonsmokers in several statuses of periodontal health: periodontally healthy, gingivitis, and periodontitis. The difference was more pronounced in gingivitis and periodontitis. The distribution of blood vessels showed that small and medium blood vessels were more common in smokers, while large blood vessels were more common in nonsmokers. In addition, the lumen of blood vessels in smokers was found to be narrower compared with nonsmokers.
